# Biallelic *SH2B3* germline variants are associated with a neonatal myeloproliferative disease and multisystemic involvement

**DOI:** 10.1038/s41431-025-01877-y

**Published:** 2025-06-06

**Authors:** Davide Leardini, Elisabetta Flex, Elliot Stieglitz, Sara Cerasi, Salvatore Nicola Bertuccio, Francesco Baccelli, Krisztián Kállay, Paula Kjollerstrom, Sara Batalha, Giovanna Carpentieri, Lucia Pedace, Andrea Ciolfi, Mahmoud Hammad, Maria Miranda, Marta Rojas, Anupama Rao, Andrew J. Innes, Martina Rudelius, Valeria Santini, Marco Raddi, Kok-Hoi Teh, Rita De Vito, Ayami Yoshimi, Marco Tartaglia, Franco Locatelli, Charlotte M. Niemeyer, Riccardo Masetti

**Affiliations:** 1 Pediatric Hematology and Oncology, IRCCS Azienda Ospedaliero-Universitaria di Bologna, Bologna, Italy; 2 Istituto Superiore di Sanità, Department Oncology and Molecular Medicine, Rome, Italy; 3 Department of Pediatrics, Benioff Children’s Hospital, University of California, San Francisco, CA USA; 4 IRCCS Azienda Ospedaliero-Universitaria di Bologna, Bologna, Italy; 5 Department of Pediatric Haematology and Stem Cell Transplantation, Central Hospital of Southern Pest, National Institute of Hematology and Infectious Diseases, Budapest, Hungary; 6 Pediatric Hematology Unit, Hospital de Dona Estefânia, Centro Hospitalar Universitário de Lisboa Central (CHULC), Lisbon, Portugal; 7 Molecular Genetics and Functional Genomics, Ospedale Pediatrico Bambino Gesù, IRCCS, Rome, Italy; 8 Department of Onco-Hematology, Cell Therapy, Gene Therapy and Hemopoietic Transplant, Ospedale Pediatrico Bambino Gesù, IRCCS, Rome, Italy; 9 National Cancer Institute, Cairo University, Cairo, Egypt; 10 Unidad Nacional de Oncologia Pediatrica, Villa Nueva, Guatemala; 11 Department of Pediatric Hematology-Oncology, Stollery Children’s Hospital, Alberta, AB Canada; 12 Department of Hematology, Great Ormond Street Hospital NHS Foundation Trust, London, UK; 13 Department of Clinical Haematology, Imperial College Healthcare NHS Trust, Hammersmith Hospital, London, UK; 14 Institute of Pathology, Ludwigs Maximilians University, Munich, Germany; 15 MDS Unit, AOUC, University of Florence, Florence, Italy; 16 Department of Pediatrics, Hospital Tunku Azizah, Kuala Lumpur, Malaysia; 17 Pathology Unit, Department of Laboratories, Bambino Gesù Children’s Hospital, IRCCS, Rome, Italy; 18 Division of Pediatric Hematology and Oncology, Department of Pediatrics and Adolescent Medicine, Medical Center, Faculty of Medicine, University of Freiburg, Freiburg, Germany; 19 Catholic University of the Sacred Heart, Rome, Italy

**Keywords:** Haematological diseases, Genetics research

## Abstract

Known genetic disorders, such as Noonan syndrome and Down syndrome, can present in the neonatal period or early infancy with myeloproliferative disease (MPD) or abnormal myelopoiesis, which often self-resolves. This phenomenon results from an imbalance in differentiation and cell regulation caused by the genetic condition during perinatal hematopoiesis. Recently, *SH2B3* variants have also been associated with neonatal MPD. However, data on their clinical significance, particularly across the spectrum of extra-hematological manifestations, of *SH2B3* variants remain limited. Here, we describe the clinical features of ten children with SH2B3-associated disease, arising from germline biallelic *SH2B3* loss-of-function (LoF) mutations in eight patients and in two patients from monoallelic germline LoF variants with loss-of-heterozygosity in hematopoietic cells. Patients displayed a MPD in the first weeks of life, which was mostly self-limiting. Following the normalization of blood counts, thrombocytosis developed during childhood. Moreover, they presented with a multisystemic clinical features consisting in delayed growth, variable neurological impairment, autoimmune disorders. These data contribute to the definition of a clinical phenotype associated with germline biallelic *SH2B3* LoF variants presenting with neonatal MPD, with important implications for patient management and follow-up.

## Introduction

Neonatal myeloproliferative disorders (MPDs) are rare conditions associated with genetic disorders such as Noonan syndrome, primarily caused by PTPN11 variants [1], Noonan-like syndromes [2] or Down syndrome [3]. These disorders typically arise during the neonatal period or early infancy and are related to impaired cellular regulatory mechanisms driven by the underlying genetic conditions. Unlike other MPDs, neonatal MPDs are not typically associated with the acquisition of additional somatic mutations, except for the sole acquisition of GATA1 variants in trisomy 21 [3]. In most cases, these conditions resolve spontaneously and occur in the context of broader developmental alterations. Nonetheless, many neonatal MPD remains without a known cause. Recently, *SH2B3* variants, have been associated with peculiar hematological features of the early infancy. The *SH2B3* gene [OMIM 605093] encodes a member of the SH2B adaptor family of proteins, SH2B3, also known as lymphocyte adaptor protein, LNK, which functions as a negative regulator of multiple cytokine and growth factor receptor signaling pathways, including the JAK/STAT pathway [4, 5]. *SH2B3* is highly expressed in hematopoietic stem and progenitor cells, where it is involved in stem cell expansion and self-renewal, and negatively modulates erythropoiesis and megakaryopoiesis [6–8]. In hematological conditions somatic *SH2B3* variants have been described in 5–7% of patients with myeloproliferative neoplasms (MPN) and, at lower frequency, in juvenile myelomonocytic leukemia (JMML) [9, 10], high-risk B- and early T-cell precursor acute lymphoblastic leukemia (ALL) [4]. Recently, loss-of-function (LoF) germline *SH2B3* variants were reported by two independent groups in patients referred to their reference diagnostic laboratories for JMML who lacked a RAS pathway mutation [9, 10]. They identified eleven children with biallelic germline variants, presenting with peculiar MPD followed by thrombocytosis and extra-hematopoietic symptoms. This evidence shadowed some preliminary reports of four patients from three families with biallelic germline SH2B3 variants. Perez-Garcia et al. reported on a homozygous inactivating *SH2B3* variant in two siblings from a consanguineous marriage with hepatosplenomegaly, leukocytosis that normalized without interventions, anemia in the first weeks of life and subsequently developed B-precursor ALL. Both siblings had impaired growth and mild developmental delay [11]. Blombery et al. reported two non-related infants with a similar leukoerythroblastic picture but thrombocytosis [12]. Of note, the four children later developed autoimmune diseases, including chronic autoimmune hepatitis, Hashimoto thyroiditis, diabetes mellitus and alopecia areata. Germline monoallelic *SH2B3* variants were also found in some patients with familial MPN, myelodysplasia (MDS)/MPN-overlap syndrome with ring sideroblasts, chronic myelomonocytic leukemia, erythrocytosis and immune cytopenia [6, 7, 11–16]. Moreover, SH2B3 variants have been reported in extra-hematological pathological conditions [17, 18]; specifically, genome-wide studies have associated somatic *SH2B3* polymorphisms with autoimmune diseases, including multiple sclerosis [19], type 1 diabetes [17], and systemic lupus erythematosus [17]. In vitro studies demonstrated that *Sh2b3*-deficient cells exhibited increased JAK2 and STAT3 phosphorylation, indicating enhanced JAK-STAT signaling, and showed increased growth and proliferation, compared with control cells [12]. While increasing evidence is elucidating the role of germline *SH2B3* variants in various disease settings, the broader clinical phenotype remains undefined. Here, we provide a comprehensive description of the hematological and extra-hematological features supported by follow-up data in patients with biallelic germline SH2B3 variants.

## Methods

This was an international, multicenter, retrospective study on pediatric patients with germline variants in *SH2B3* (NM_005475.3, NP_005466.1). The sole inclusion criterion was the confirmed presence of a germline biallelic or monoallelic with somatic loss of heterozygosity (LOH) *SH2B3* variant, with genetic testing performed at each participating center according to the local protocols. Ten patients from nine centers worldwide were included. Consent for genetic studies and evaluation of clinical data for research had been obtained from patients’ guardians according to local procedures. The study design was approved by the Ethical Committee CE-AVEC (code 656/2024/Oss/AOUBo). Clinical information, including disease course and treatment responses, was reported as assessed by the local treating physicians. Bone marrow (BM) examinations, including assessments of BM cellularity, were conducted following local practices. Hematological disorders were classified according to the International Consensus Classification of Myeloid Neoplasms [20]. To examine the structural context of affected residues, UCSF Chimera software (https://www.cgl.ucsf.edu/chimera/) [21] was used in combination with the crystallographic structure of the murine Sh2b3 SH2 domain complexed with the JAK2 pY813 motif (PBD reference: 7r8w).

## Results

We report on ten patients with germline *SH2B3* variants; preliminary data from two patients (P4.1, P5.1) were included in a previous report [10]. *SH2B3* variants were identified via clinical NGS assay, on different testing material according to centers’ practice as reported in Table 1. The median age at presentation of the six females and four males was 0.2 years (range 0–4.0 years). One patient was lost to follow-up shortly after diagnosis; the median time to follow-up of the remaining patients was 5.0 years (range, 0.7–30.0 years).

**Table 1:** Mutation, hematological features, and therapy.

ID	Mutation				MPD									Thrombocytosis			
	Location	Germline	Tested specimen	Blood/BM	Age (y)	WBC (x10^9^/L)	% Blasts (PB)	Myeloid/erythroid precursors (PB)	Platelets (x10^9^/L)	% Blasts (BM)	Karyotype (BM)	Splenomegaly/ Hepatomegaly	Therapy (months)	Age (y) first noted	Platelets (x10^9/^L) at diagnosis	Splenomegaly	Therapy (years)
P1.1	p.Arg392Trp	Homo	Hair follicle	Homo	0.1	93	6	+	121	7	Normal	+	Aza, splenectomy (6), HSCT (7)	Not evaluable following splenectomy/HSCT			
P1.2	p.Arg392Trp	Homo	Hair follicle	Homo	0	101	2	+	35	2	Normal	+	Aza, ruxo, ven, splenectomy (4), HSCT (9)	Not evaluable following splenectomy/HSCT			
P2.1	p.Asp570Lysfs^b^82	Homo	Buccal swab	Homo	0	No data	11	+	19	No data	Normal	+	none	8.0	700	+	HSCT^a^(10)
P4.1	p.Leu438Arg	Homo	Hair follicle	Homo	0	25	3	+	33	9	Normal	+	Cytarabine, 6-MP	0.8	862	+	none
P5.1	p.Arg392Gln	Homo	Hair follicle	Homo	0.4	102	5	+	75	4	Normal	+	HSCT (16)	No thrombocytosis prior to HSCT			
P6.1	p.Lys278^b^	Homo	Hair follicle	Homo	0.1	170	0	+	46	2	Normal	+	6-MP	0.2	770	+	6-MP
P8.1	p.Lys278Argfs^b^2	Hetero	Hair follicle	Homo	0.2	46	4	+	606	0	Normal	+	none	0.2	606^b^ (1100 at 4 y of age)	+	none
P9.1	p.Gly387^b^	Hetero	Hair follicle	Homo	0.7	36.9	2	+	391	1	Normal	+	none	Lost to follow-up after initial presentation			
P3.1	p.Met1Val	Homo	Hair follicle	Homo	Not presented MPD									0.3	1160	−	none
P7.1	p.Glu400Lys p.Glu395Lys	Compound hetero	Skin fibroblasts	Homo	Not presented MPD									4.0	1077	+	none

*Aza* azacytidine, *BM* bone marrow, *HSCT* hematopoietic stem cell transplantation, *6-MP* 6-mercaptopurine, *MPD* myeloproliferative disease, *PB* peripheral blood, *ruxo* ruxolitinib, *ven* venetoclax, *WBC* white blood cells, *Y* year.

^a^Patient was transplanted for myelofibrosis grade 3.

^b^Platelet count at initial presentation, see also under MPD.

### Genetic mutational landscape

**Fig. 1:**
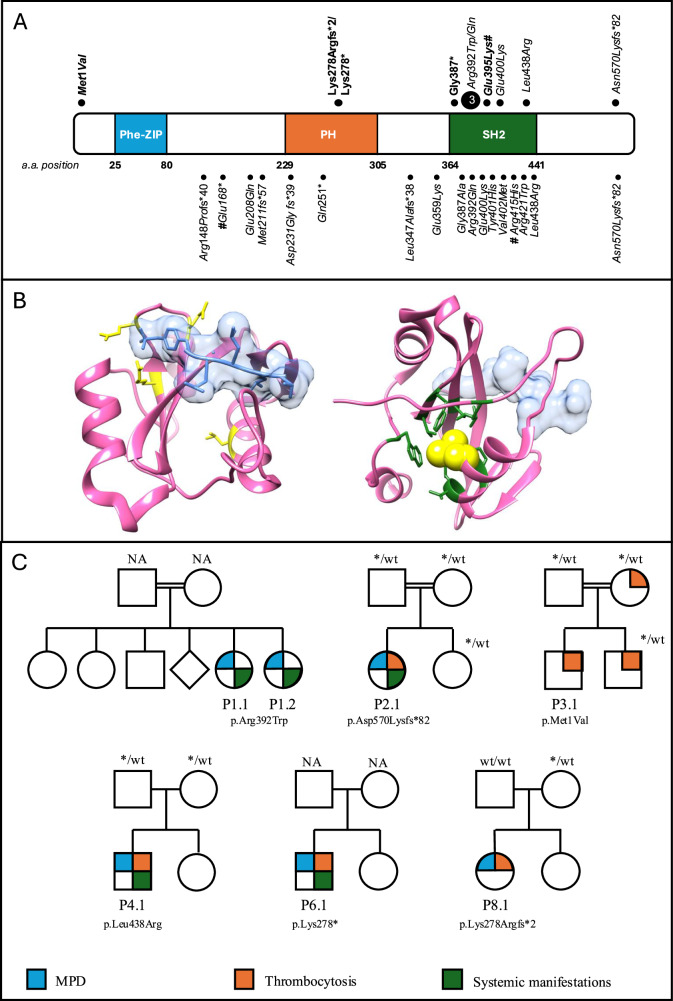
Genetic and structural characterization of SH2B3 germline variants in the study cohort. **A** The upper section shows the distribution of SH2B3 variants in the study cohort on the SH2B3 protein. Mutations in bold have not been previously reported. The lower section presents published SH2B3 germline variants. PH: Pleckstrin homology domain; Phe-ZIP: Phenylalanine zipper; SH2: SH2 domain. #Mutation in trans on IGV variant viewer. **B** Location of Arg392, Glu395, Glu400, and Leu438 within the SH2 domain of SH2B3. The panel shows the SH2 domain (pink) complexed with the JAK2 peptide containing pTyr813 (light blue) (PBD: 7r8w). Three residues (Arg392, Glu395, and Glu400) are located close to pTyr813 and their non-conservative substitutions are predicted to perturb the intermolecular binding network stabilizing the SH2B3-JAK2 interaction (left). The mutated residues are highlighted in yellow with their lateral chains. The lateral chain of Leu438 is placed in a buried pocked formed by several hydrophobic residues (Trp364, Phe389, Val391, Leu402, Val434, Val435, Met437, Leu458; lateral chains showed in green). The Leu-to-Arg substitution introduces a positively charged lateral chain that is expected to dramatically perturb the conformational organization of the region, likely resulting in an aberrant folding of the entire SH2 domain (right). Both events are predicted to result in a defective function of the SH2 domain, causingAQ9 impaired binding of SH2B3 to JAK, and failure to functionally downmodulate the kinase. **C** Pedigrees for seven patients. Individual with a homozygous mutation or compound heterozygous or with a homozygous mutation on hematopoietic stem cells in each family are marked with arrows. Double line indicates consanguinity, which is present in three families. MPD myeloproliferative disorder; NA not assessed; wt wild-type; * SH2B3 variant.

Eight patients were homozygous or compound heterozygous for germline *SH2B3* variants, two were heterozygous for germline *SH2B3* variants with homozygous variants in hematopoietic cells. All SH2B3 variants were private/rare (Fig. 1A, Supplementary Table S1), five variants (p.Glu395Lys, p.Lys278Argfs*2, p.Lys278*, p.Gly387*, p.Met1Val) have not been previously reported (Fig. 1A), and three of the families were consanguineous. Among the identified variants, one was a start-loss single nucleotide substitution affecting the initiation codon of the transcript (p.Met1Val), four were frameshift (p.Lys278Argfs*2, p.Lys278*, p.Gly387*, p.Asn570Lysfs*82), and five were missense specifically affecting the SH2 domain of the protein (p.Arg392Trp, p.Arg392Gln, p.Glu395Lys, p.Glu400Lys, p.Leu438Arg), a region largely intolerant to missense variation [22]. Consistently, all variants were classified as likely pathogenic, with the exception of p.Glu400Lys and p.Leu438Arg that were classified as variant of uncertain significance (VUS). Structural inspection of the location of affected residues performed as specified in the Methods [21] showed that Arg392, Glu395, and Glu400 are located close to the phosphorylated Tyr813 residue, and their non-conservative substitutions were predicted to perturb the intermolecular binding network stabilizing SH2B3 binding to phosphorylated JAK2 (Fig. 1B) [8]. Similarly, the lateral chain of Leu438 points to a buried pocket formed by a number of hydrophobic residues (Trp364, Phe389, Val391, Leu402, Val434, Val435, Met437, and Leu458). The Leu-to-Arg change at codon 438 was predicted to disrupt the structure of this region possibly perturbing proper folding of the SH2 domain. These considerations point to a mechanism for all the identified amino acid changes involving a defective function of the SH2 domain, resulting in impaired binding of SH2B3 to JAK and its functional downmodulation. Consistent with a LoF model, frameshift variants were spotted throughout the coding sequence of *SH2B3*. A somatic mutation in *SETBP1* (c.1178_1179ins59) at a low variant allele frequency (VAF) of 4% in P 2.1, and a germline VUS in NF1 (c.7915C > G) at a VAF of 50% in P 9.1 were noted in the cohort.

### Hematological presentation

**Fig. 2:**
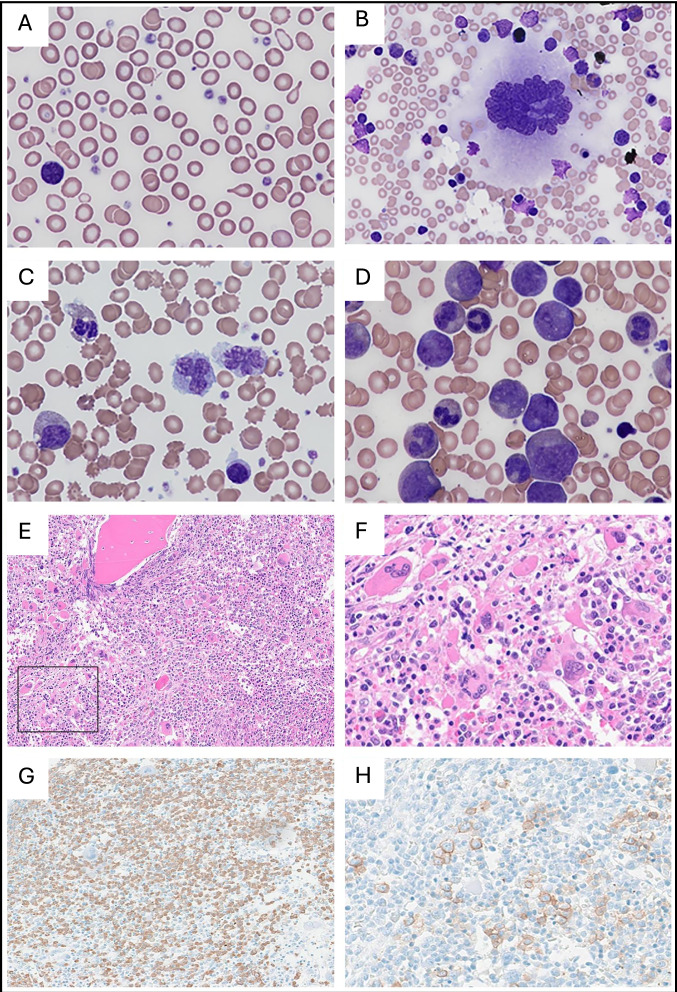
Peripheral blood and bone marrow findings in patients with SH2B3 germline variants. Peripheral blood **A**, **C** and BM aspirate **B**, **D** of P8.1 **A**, **B** and P9.1 **C**, **D**: Anisopoikilocytosis of red blood cells with teardrops, increased platelets with anisocytosis and giant platelets **A** Enlarged megakaryocyte with abundant cytoplasm and hyperlobulated nucleus **B**. Myelocyte and atypical monocytes. **C** Hyperplastic and left shifted myelopoiesis **D**. BM biopsy of patient P1.1 **E**–**H**: Hypercellular BM **E**, **H**, **E** with clusters of atypical, enlarged megakaryocytes (**F**, (**H**, **E**; 4x magnification of **E**). Increased granulopoiesis (**G**; MPO immunohistochemistry) and reduced and left shifted erythropoiesis (**H**; CD71 immunohistochemistry).

Of the ten patients studied, eight presented with a MPD characterized by leukocytosis with a leukoerythoblastic picture, low blast percentage in BM and splenomegaly, at birth or in the first months of life (Table 1). Median white blood cell count at presentation was 93 × 10^9^/L (range 25–170) and median monocyte count 9.2 × 10^9^/L (range 3.2–18.1). Immature myeloid and erythroid cells on peripheral blood smear were noted in all patients, while six patients had circulating blast cells. Karyotype was on BM was normal for all patients. The platelet count at the time of MPD diagnosis varied widely among the eight patients, with values ranging from 19 × 10⁹/L to 606 × 10⁹/L. Six patients presented with thrombocytopenia with a median value of 40.5 × 10⁹/L (range 19–121), while one had normal platelet levels (391 × 10⁹/L), and one had thrombocytosis (606 × 10⁹/L). Two patients had a slightly elevated blast percentage in the BM, namely, 7 and 9%. Cytoreductive therapy for MPD was administered to four of the eight patients, which included treatments such as azacytidine, ruxolitinib, venetoclax, 6-mercaptopurine, and two patients underwent splenectomy for rapidly enlarging spleen size. Two patients (P1.1 and P1.2) were treated with allogeneic stem cell transplantation (HSCT) for non-response of myeloproliferation to anti-leukemic agents, one patient (P5.1) for a presumed diagnosis of JMML, and one patient for myelofibrosis at 10 years of age (Table 1). Overall, all patients experienced a complete remission of the MPD. Three of the six patients with MPD and thrombocytopenia (P2.1, P4.1, P6.1) were evaluable for the natural course of their blood counts, which normalized within a few months. However, these patients later developed persistent thrombocytosis at 0.2, 0.8, and 8.0 years of age, with platelet counts ranging from 700 × 10⁹/L to 862 × 10⁹/L (Table 1, Supplementary Fig. S1). In addition, two of the ten patients in this cohort (P3.1, P7.1), who did not present with MPD, displayed isolated, as the sole hematological feature, persistent thrombocytosis diagnosed at 0.3 and 4.0 years of age, with platelet counts of 1160 × 10⁹/L and 1077 × 10⁹/L, respectively. These two patients were diagnosed during routine blood tests. None of the 6 patients with patients with thrombocytosis experienced symptoms associated or required specific treatments for this platelet elevation. On peripheral blood smear, there was marked anisocytosis of platelets with giant platelets (Fig. 2A, C). Leukocytosis with atypical monocytosis was present and the percentage of eosinophils was often elevated. Myeloid, as well as erythroid, precursors were noted. BM aspirate showed an increased number of megakaryocytes with enlarged and abundant cytoplasm and hyperlobulated nuclei (Fig. 2B, D). Myelopoiesis was hyperplastic and left shifted. Erythropoiesis was hypoplastic without significant dysplasia. BM biopsies were performed during the initial diagnostic assessment in three patients (P1.1, P1.2, P2.1). Morphological analysis revealed a hypercellular marrow with hyperplastic megakaryopoiesis forming large clusters (Fig. 2E–H). The megakaryocytes themselves appeared atypical and polymorphic with a prevalence of enlarged and hyperlobulated cells. Granulopoiesis was increased and left-shifted with a slight increase in eosinophils. Erythropoiesis was significantly reduced with an increase in immature precursors. There was neither evidence of BM fibrosis nor significant proliferation of monocytes or myeloblasts.

### Extra-hematological features and family study

Most of the patients presented with extra-hematological features (Table 2). Eight patients presented with intrauterine growth restriction (IUGR) or were small for gestational age, and two patients had been delivered pre-term. Median birth weight was 2000 g (range, 1400–3570). One patient (P8.1) presented with severe cognitive impairment. Six others had developmental delay; among the four children who underwent formal psychological assessment, three (P2.1, P4.1, P5.1) had normal results, while one (P6.1) showed mild cognitive impairment. Five patients showed dysmorphic features, which included low-set, posteriorly rotated ears, a prominent forehead with a high hairline, mild hypertelorism, absence of one finger in the right hand, and microcephaly. For the two patients with microcephaly (P5.1 and P6.1), the condition was not present at birth but was noted during growth monitoring. Two patients developed autoimmune diseases during childhood at a median age of 10 years (range 9–11). Nine relatives of six index cases were studied for *SH2B3* variants (Fig. 1C**)**. Two siblings of index cases presented monoallelic *SH2B3* variants; one (sister of P2.1) was free of symptoms, while the other (brother of P3.1) had moderate thrombocytosis (value of 500 × 10^9^/L) since birth. Among the five parents carrying monoallelic germline variants, only one subject (mother of P3.1) had persisting JAK2-negative thrombocytosis since the age of 15 years and Crohn’s disease with arthritis and vitiligo (value of >1.000 × 10^9^/L in more than one determination, normal the rest of complete blood count). The other parents were asymptomatic with normal blood counts.

**Table 2:** Extra-hematological features.

ID	Geographic region	Consanguineous parents	Sex	Birth’s week	Weight at birth (g)	IUGR /SGA	Growth retardation /small stature	Dysmorphic features	DD/cognitive impairment	Autoimmune manifestation (y)	Follow-up (age in years)
P1.1	HU	Yes	F	38	1490	+	+	Low-set and posteriorly rotated ears; prominent forehead with high hair line, mild hypertelorism	Speech and walking delay	−	Alive (1.9)
P1.2	HU	Yes	F	40	2280	+	+	Same than P1.1	Same than P1.1	−	Alive (0.7)
P2.1	ITA	Yes	F	36	2000	+	+	−	Speech and walking delay, no cognitive impairment	Multiple sclerosis (9) Diabetes mellitus (10)	Alive (14.4)
P4.1	CAN	Unknown (parents from the same indigenous reserve)	M	33	1490	+	−	Absence of one finger in the right hand	Speech delay	−	Alive (5.0)
P5.1	EGY	Yes	F	40	1800	+	+	Microcephaly	Speech and walking delay	−	Alive (1.7)
P6.1	GBR	Unknown	M	40	2010	+	+	Microcephaly	Mild global developmental delay and cognitive impairment	Autoimmune hypothyroidism (10) Localized scleroderma (11)	Alive (15.0)
P8.1	MYS	Unknown	F	40	1400	+	+	−	Severe cognitive impairment, autism	−	Alive (4.0)
P9.1	GTM	Unknown	F	No data	No data	No data	−	−	−	Lost to follow-up	
P3.1	PRT	No	M	39	3570	−	−	−	−	−	Alive (12.0)
P7.1	GBR	No	M	42	2700	+	−	−	−	−	Alive (34.0)

*CAN* Canada, *DD* developmental delay, *EGY* Egypt, *GBR* Great Britain, *GTM* Guatemala, *HU* Hungary, *ITA* Italy, *IUGR* intrauterine growth restriction, *MRI* magnetic resonance imaging, *MYS* Malaysia, *PRT* Portugal, *SGA* small for gestational age.

## Discussion

We here describe the clinical features of a cohort of 10 patients with germline *SH2B3* variants. In our cohort, biallelic *SH2B3* variants were associated in eight patients with MPD in the first weeks of life, including two with a monoallelic germline variant who acquired somatic LOH. Considering the age of onset of the phenotype, respectively at 0.2 and 0.7 years, the two patients most likely acquired LOH for the SH2B3 variant pre- or early postnatally and thus it is conceivable that the observed phenotype is related to the biallelic alteration of the gene. This suggest that the LOH occurred through a uniparental disomy (UPD) mechanism or a gene loss during early hematopoiesis, may lead to clonal expansion of hematopoietic cells. Interestingly, the VAF in blood for patient P8.1 who carries a monoallelic variant in germline material, was close to 100%, suggesting a near-complete replacement of normal hematopoiesis by the mutant clone. Unfortunately, detailed VAF dynamics pre- and post-treatment were not available for this patient. However, tracking changes in VAF over time could provide valuable insights into the clonal dynamics and the extent to which normal hematopoiesis can recover following the resolution of MPD. This observation is particularly novel and has significant implications for understanding the pathogenesis of SH2B3-related disorders. The mechanism of LOH is reminiscent of what is commonly observed in JMML, where somatic or acquired UPD is a well-recognized driver of clonal dominance. Future studies investigating the role of similar LOH mechanisms in SH2B3-related disorders may uncover parallels to other hematopoietic conditions and improve our understanding of disease progression and variability in clinical phenotype.

The clinical course is characterized by leukoerythroblastosis, monocytosis, low blast percentage in blood and marrow, splenomegaly, a normal karyotype and absence of somatic mutations. This presentation of the constitutional SH2B3-related disease is reminiscent of that observed in other MPD in neonates or infants, such as in patients with Down syndrome with somatic mutations in *GATA1*, or in RASopathies associated with germline mutations in *CBL* or *PTPN11*. In particular, MPD in CBL syndrome characterized by monoallelic germline mutation and LOH in hematopoietic cells, runs a self-resolving clinical course in the vast majority of cases. Interestingly, SH2B3 recruits the CBL protein via its conserved C-terminal tyrosine residue leading to an interaction between JAK-STAT and RAS-MAPK signaling. This interaction suggests that SH2B3 loss-of-function promotes myeloproliferation by activating the RAS-MAPK pathway through altered CBL modulation [2, 23, 24]. This was confirmed in transgenic mouse models, where the loss of both Lnk and Cbl leads to severe splenomegaly, extramedullary hematopoiesis, and exacerbated myeloproliferative characteristics [25] and reported in a previous case report [15]. Like in previous reports [9, 11, 12], the clinical course of neonatal MPD in patients with *SH2B3* germline disease presented here was self-limiting in most cases. Three patients did not receive any therapy; two patients were treated with low dose cytoreductive therapy to ameliorate myeloproliferation. In two patients, chemotherapy and BCL-2 inhibition failed to control the disease, suggesting that some cases of SH2B3-related neonatal MPD may not run an indolent course. Considering the preclinical evidence of JAK-inhibition in *SH2B3*-mutant cells [10, 12], ruxolitinib could be an attractive therapy option. In the previous report ruxolitinib was effective in the resolution of splenomegaly and in the reduction of SH2B3 variant allele frequency [10]. In our cohort, ruxolitinib did not show efficacy in the treated patient. All patients in our cohort were alive at the last follow-up, including the three who received allogeneic HSCT, and none presented with abnormal blood counts except for thrombocytosis.

Indeed, the clinical presentation of these MPD also resembles JMML. Not surprisingly, SH2B3 variants have been identified in neonates suspected of JMML who lack a RAS pathway mutation, as reported in previous studies [9, 10]. The challenges and inconsistencies in categorizing these unique MPD in young children are further highlighted by the fact that MPD in CBL syndrome is traditionally classified as JMML. Arfeuille et al. reported on eight such patients from five families carrying biallelic germline variants [9]. Notably, while in our cohort the clinical phenotype shared features with JMML, the morphology, in particular the prominent atypical megakaryopoiesis and the absence of a significant increase in monocytic cell forms, was not characteristic of JMML [26]. However, since we evaluated BM specimens obtained in early infancy and not in the neonatal period, it is conceivable that the number of megakaryocytes in affected newborns is reduced as described by the French investigators [9], and the dysplastic features described arise later in early infancy.

In our cohort, following the resolution of MPD, patients with biallelic germline disease developed persistent thrombocytosis. One of these children underwent allogeneic HSCT for myelofibrosis at 10 years of age. In two other patients in this cohort (P 3.1, P 7.1), thrombocytosis diagnosed at 0.3 years and 4 years of age was the sole initial hematological presentation. Interestingly, the two children with monoallelic germline variants and LOH in hematopoietic cells had normal or moderately elevated platelet counts when neonatal MPD was diagnosed. The observation of thrombocytosis is consistent with the role of SH2B3 as a negative regulator of JAK/STAT signaling as previously demonstrated [4, 10, 12], also demonstrated by the increase in megakaryocytic progenitors, megakaryocytes, and platelets in hematopoietic tissue, along with an increase in erythroid progenitors, reported in *Sh2b3*-deficient mice [27]. Notably, the impact of SH2B3 LoF appears to be age-dependent with features of MPD in newborns and young infants and isolated thrombocytosis later in childhood, suggesting that *SH2B3* variants have different effects on fetal and adult hematopoiesis. Remarkably, the mother of patient 3.1, who carries a heterozygous germline variant, has been known to have thrombocytosis since adolescence. Considering the clinical phenotypes associated with germline *SH2B3* variants, it is puzzling that they can cause thrombocytosis or erythrocytosis in the absence of somatic mutations [28], while also cooperating with other driver mutations such as JAK2 or CALR to result in adult-type MPD [6, 7, 13, 14, 29], or PTPN11 to result in JMML. It must be said that one patient in out cohort presented a germline VUS in NF1; however, the patient did not develop any sign or symptoms of NF1-related conditions.

Given the lack of robust data on the long-term outcomes of this rare hematological disorder, a watch-and-wait strategy appears to be a reasonable initial approach, as suggested by others [9, 10]. In cases of extreme leukocytosis with pulmonary complications or significant organomegaly, cytoreductive therapy with 6-mercaptopurine or low-dose cytarabine may provide symptomatic relief. Later in the clinical course, disturbed megakaryopoiesis may lead to progressive myelofibrosis, resulting in an indication for HSCT.

Previous reports have described associations between germline *SH2B3* variants and specific autoimmune conditions [9, 10, 17, 30]. In the presented cohort, six patients with biallelic *SH2B3* germline variants and one of the two patients with a monoallelic germline variant and LOH in hematopoietic tissue displayed extra-hematopoietic symptoms, including IUGR, developmental delay, growth retardation, and dysmorphic facial features. Indeed, IUGR appears to be the most consistent constitutional phenotype. The underlying mechanism of this delay remains unclear, but one possibility is that it represents a hypoproliferative phenotype, potentially driven by an embryonic effect of SH2B3. To explore this, we examined available complete blood count data at birth in two patients who later developed MPD (P1.1 and P1.2), both of whom had normal hemoglobin levels. This very preliminary finding suggests that the phenotype may be more consistent with an intrinsic hypoproliferative mechanism rather than anemia-induced growth restriction. The presence of extra-hematological pathological features in both monoallelic and biallelic variant patients, suggest that a single hit may be sufficient to produce a phenotype. Indeed, the high incidence of these abnormalities in families with consanguineous parents may be a confounding factor, and larger cohorts will help define these clinical features [12]. Arfeuille et al. reported extra-hematological features as well, including cardiac involvement, which we did not confirm in ours [9]. Our report also underlines the frequent development of autoimmunity manifesting itself in childhood [9, 11, 12]. The role of SH2B3 in autoimmunity is suggested by earlier genome-wide studies in a variety of autoimmune disorders like rheumatoid arthritis, coeliac disease, hepatitis or diabetes type 1 [19, 31–35]. Moreover, it was shown that *SH2B*3 variants in patients with systemic lupus erythematosus are predominantly hypomorphic alleles failing to suppress interferon type II signaling via JAK2-STAT1 and impairing the negative selection of self-reactive B cells in mice [30]. This evidence may explain the high incidence of autoimmunity in these patients. However, further functional in vivo studies, including the exploration of biological markers related to cellular and humoral immunity, will be necessary to better characterize potential immune deregulations that contribute to their susceptibility to autoimmune diseases in late childhood. Apart from a distinct hematological phenotype, there are overlapping extra-hematopoietic features such as IUGR, growth retardation, and developmental delay. However, dysmorphic features appear more variable among individuals and larger cohorts will be essential clarify whether these findings define a consistent syndrome.

To summarize, germline *SH2B3* LoF variants define a novel condition characterized by neonatal or in early infancy MPD which appears to be associated with extra-hematopoietic symptoms. Awareness of this genetic condition is key to a correct diagnosis and avoidance of unnecessary intensive therapy for the MPD. The establishment of large international registries spanning all age groups will be essential to better characterize the natural history of germline SH2B3 disease and to enhance our understanding of SH2B3 function across different ages and hematological manifestations.

## Supplementary information

Supplementary Material

## Data Availability

The original data of the current study are available from the corresponding author on reasonable request.
