# Supplementary material for: Biallelic SH2B3 germline variants are associated with a neonatal myeloproliferative disease and multisystemic involvement
Source: Eur J Hum Genet. 2025 Jun 6;33(9):1127–35. doi: 10.1038/s41431-025-01877-y (PMC12402439; doi:10.1038/s41431-025-01877-y)
Supplement: Supplementary file 1 — Supplementary Material [file 41431_2025_1877_MOESM1_ESM.docx]

**BIALLELIC *SH2B3* GERMLINE VARIANTS ARE ASSOCIATED WITH A NEONATAL MYELOPROLIFERATIVE DISEASE AND MULTISYSTEMIC INVOLVEMENT**

Davide Leardini*^1^, Elisabetta Flex^2^, Elliot Stieglitz^3^, Sara Cerasi^1^, Salvatore Nicola Bertuccio^4^, Francesco Baccelli^1^, Krisztián Kállay^5^, Paula Kjollerstrom^6^, Sara Batalha^6^, Giovanna Carpentieri^7^, Lucia Pedace^8^, Andrea Ciolfi^7^, Mahmoud Hammad^9^, Maria Miranda^10^, Marta Rojas^11^, Anupama Rao^12^, Andrew J. Innes^13^, Martina Rudelius^14^, Valeria Santini^15^, Marco Raddi^15^, Kok-Hoi Teh^16^, Rita De Vito^17^, Ayami Yoshimi^18^, Marco Tartaglia^7^, Franco Locatelli^8,19^, Charlotte M. Niemeyer^18^, Riccardo Masetti^1^

**SUPPLEMENTARY MATERIAL**

**Supplementary Table S1:** Germline mutations of the ten patients included in the cohort presented.

**Supplementary Figure S1:** Temporal trend of platelets for the patients included in the study.

**Supplementary Table S1:** Germline mutations of the ten patients included in the cohort presented. ^+^ compound heterozygous, *in-trans* on IGV variant viewer. Variants reported for the first time are bolded. GnomAD v4.1.0 and GnomAD v3.1.2 (non-cancer).

| **ID** | **Genome**  **hg19** | **mRNA**  **NM_005475.3** | **Protein** | **dbSNP** | **Allele Frequency**  **GnomAD_exomes (GnomAD version v.4.1.0)** | **Allele Frequency gnomAD v3.1.2**  **(non-cancer)** |
| --- | --- | --- | --- | --- | --- | --- |
| P1.1 | chr12:111447482 | c.1174C>T | p.Arg392Trp | rs770836648 | 0.000006417 | Variant not found |
| P1.2 | chr12:111447482 | c.1174C>T | p.Arg392Trp | rs770836648 | 0.000006417 | Variant not found |
| P2.1 | chr12: 111446947 | c.1709>dupA | p.Asp570Lysfs*82 | N/A | N/A | N/A |
| P4.1 | chr 12:111447732 | c.1313 T>G | p.Leu438Arg | rs1458654949 | N/A | N/A |
| P5.1 | chr 12:111447483 | c.1175 G>A | p.Arg392Gln | rs953911126 | 0.000005474 | 0.00002034 |
| P6.1 | chr12:111446852 | **c.832 A>T** | **p.Lys278*** | N/A | N/A | N/A |
| P8.1 | chr12:111446853 | **c.833delA** | **p.Lys278Argfs*2** | N/A | N/A | N/A |
| P9.1 | chr12: 111447466 | **c.1159 G>T** | **p.Gly387*** | N/A | N/A | N/A |
| P3.1 | chr12:111418146 | **c.1A>G** | **p.Met1Val** | rs376261237 | 0.000001446 | Variant not found |
| P7.1 | chr12:111447506  chr12:111447491 | c.1198 G>A  **c.1183 G>A** | p.Glu400Lys^+^ **p.Glu395Lys^+^** | rs72650673  rs148636776 | 0.001510  0.0003621 | 0.0007838  0.0002007 |

**Supplementary Figure S1:** Temporal trend of platelets for the patients included in the study. Data were retrieved from medical records. Graphical presentation was performed with R studio software version 2022.12.0+353 (2022.12.0+353).
